# Laves Phase Evolution in China Low-Activation Martensitic (CLAM) Steel during Long-Term Aging at 550 °C

**DOI:** 10.3390/ma13010154

**Published:** 2019-12-31

**Authors:** Lie Yang, Fei Zhao, Wenyuan Ding

**Affiliations:** 1 College of Materials and Metallurgy, Guizhou University, Guiyang 550025, China; tyl19950124@163.com (L.Y.); 18300862205@163.com (W.D.); 2 Key Laboratory for Materials Structure and Strength of Guizhou Province, Guiyang 550025, China

**Keywords:** CLAM steel, Long-term aging, laves phase, precipitation

## Abstract

To clarify the precipitation and evolution law of the Laves phase in China low-activation martensitic (CLAM) steel during long-term aging at high temperature, this paper carried out an aging treatment of CLAM steel at 550 °C for up to 30,000 h. The segregation behavior of alloy elements and the precipitation amount and average size of the Laves phase were quantitatively characterized by transmission electron microscopy (TEM) and scanning electron microscopy (SEM), and the precipitation and coarsening behavior of the Laves phase were obtained. The results show that the Laves phase begins to precipitate within 5000 h after aging and mainly depends on M_23_C_6_ carbides to nucleate and grow at the grain boundary and subgrain boundary. During the aging process, the average size of the Laves phase grows continuously. After more than 25,000 h, the growth rate of the Laves phase decreases. After 30,000 h of aging, the average size reaches 439.9 nm, and the maximum size exceeds 800 nm. The area fraction of the Laves phase increases continuously during the 20,000 h aging process and tends to be stable after aging for 20,000 h. The area fraction is approximately 1.85%.

## 1. Introduction

China low-activation martensitic (CLAM) steel is a type of reduced activation ferritic/martensitic (RAFM) steel independently developed in China [1,2], whose mechanical properties, physical properties, and radiation resistance are similar to those of mainstream RAFM steels such as Eurofer97 [3,4] and F82H [5]. CLAM steel has been recognized as a candidate structural material for Chinese fusion engineering experimental reactors and has been the focus of relevant research institutes and universities in China [6]. Many studies have reported that the excellent high-temperature mechanical properties of RAFM steel can be attributed to the dispersion strengthening of precipitates, including M_23_C_6_ and MX (M-Ta, M-C or N) precipitates originally existing in RAFM steel, and of course, the Laves phase [7,8,9].

However, there are some different opinions on the effect of the Laves phase on the high-temperature mechanical properties of martensitic steel [10,11]. Some studies have reported that the nucleation of the Laves phase promotes the dissolution of W, reduces the thermal stability of the microstructure, and causes a decrease in the strength [12,13,14]. At the same time, studies on the creep strength of martensitic steel found that the coarsened Laves phase facilitate void growth and cause brittle intergranular fractures [7,15]. Hence, it is believed that the nucleation and coarsening of the Laves phase greatly weakens the stability of the microstructure, which is detrimental to the high-temperature mechanical properties of the material. However, Abe [16] found that the initially formed fine Laves phase can reduce the creep rate, when the Laves phase is coarsened, the creep rate is increased. To correctly evaluate the high-temperature microstructure and mechanical property stability of RAFM steels, Japan conducted a thermal aging study of 100,000 h at 400–650 °C for F82H [17] steel, analyzed the evolution of microstructural and mechanical properties, and mastered the evolution of the Laves phase. It provided an effective reference for RAFM steels. However, there are some differences in the content of the main alloying elements between F82H steel and CLAM steel, the results of F82H steel cannot be simply applied to CLAM steel.

Recently, some studies on the Laves phase of CLAM steel have been completed in the service environment of simulated fusion reactors. Yang et al. [18] did not find nucleation of the Laves phase after aging for 3000 h at 600 °C, it is known that the lower W content delays the precipitation time of the Laves phase and enhances the stability of the microstructure. In a recent study, Wang et al. [19] found that the Laves phase was formed at the grain boundary after aging for 10,000 h at 550 °C, and the coarsened M_23_C_6_ carbide was considered to be the factor for the increase of the ductile-brittle transition temperature (DBTT). The effect of the Laves phase is overlooked. However, Huang [20] et al. and Hu [21] et al. found that the coarsened Laves phase reduces the grain boundary binding force, which is the important factor for the increase in DBTT. 

At the same time, a study on the long-term creep of CLAM steel found that the creep stress enhanced the formation and growth of the Laves phase [22], and that the creep stress will be the factor of breakdown of creep strength [23]. Although these studies have made some progress, they remain insufficiently systematic due to short aging times. The precipitation mechanism and growth mechanism of the Laves phase in CLAM steel during long-term aging has rarely been reported in the literature. Therefore, studies on the long-term aging of CLAM steel must be conducted to systematically study the evolution of the Laves phase. Only in this way can the high-temperature structural stability of CLAM steel be accurately evaluated.

The maximum service temperature of CLAM steel used in Chinese fusion engineering experimental reactors is recommended to be 550 °C. Therefore, long-term thermal aging at 550 °C can correctly evaluate the stability of CLAM steel during service. In the present work, based on the study of F82H steel aging for 100,000 h, the aging treatment of CLAM steel for 30,000 h was carried out at 550 °C to study the evolution of the Laves phase and provide theoretical guidance for engineering applications.

## 2. Material and Experimental Procedure

The studied CLAM steel was melted by vacuum induction technology, and then the electroslag was remelted to obtain a 500 kg ingot. Finally, the ingot was forged (the initial forging temperature was 1100–1150 °C, and the final forging temperature was higher than 850 °C) and hot-rolled (completely recrystallized + non-recrystallized zone) into a 21 mm thickness plate. The heat treatment parameters of CLAM steel were: normalized at 980 °C for 30 min, and then tempered at 760 °C for 90 min with air-cooling after each stage. The tempered CLAM steel plate was cut into strip samples (60 mm × 10 mm × 10 mm) along the rolling direction by a wire electric discharge machine and placed into a vacuum quartz tube for sealing. Finally, the aging treatment was conducted in a box furnace at 550 °C for 5000 h, 10,000 h, 15,000 h, 20,000 h, 25,000 h, and 30,000 h. The chemical composition of CLAM steel is shown in Table 1.

After aging for different times, the samples were abraded with emery paper and then polished with cloth. All polished samples were etched using Vilella’s (5 g FeCl_3_ + 50 mL HCl + 100 mL H_2_O) reagent at room temperature for 1 min. The evolution of precipitates during long-term aging was studied by thermal field emission scanning electron microscopy (SEM) with a voltage of 10 kV (Zeiss Supra 40, Oberkochen, Germany). Energy dispersive spectroscopy (EDS) (Oxford Aztec 3.1, London, UK) was utilized to determine the principal alloying element concentration before and after aging heat treatment (to make the regularity of precipitates more obvious, selecting aged samples of 0, 10,000 h, 20,000 h, and 30,000 h). Then, according to the difference of the average atomic number, the Laves phase and M_23_C_6_ carbides in all aged samples were discerned by the backscattered electron (BSE) modes of SEM with a voltage of 15 kV (Hitachi SU8020, Tokyo, Japan). The Laves phase will be bright due to the high average atomic number. 

We quantitatively determined the size and area fractions of the Laves phase using Image-Pro Plus. In this work, at least four images were analyzed at 5000× magnification. All samples for transmission electron microscopy (TEM) (FEI Tecnai G2 F20, Hillsboro, OR, USA) were prepared. Firstly, 0.6 mm thickness slices were cut from the aged and tempered samples by a wire electric discharge machine, followed by emery paper polished down to below 100 µm. Finally, Ø 3 mm discs were punched from the above slices and thinned to the final thickness with an ion thinning instrument (Gantan691, Hillsboro, OR, USA). The nucleation mechanism, the type, and the chemical composition of the Laves phase were performed by TEM with selected area electron diffraction (SAED) and EDS.

## 3. Results and Discussion

### 3.1. Long-Term Aging Effect on Microstructure and Precipitates

**Figure 1:**
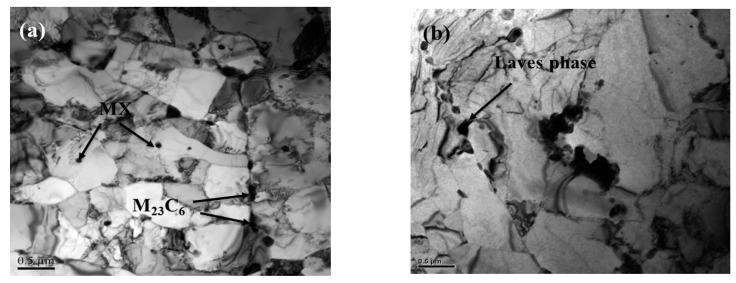

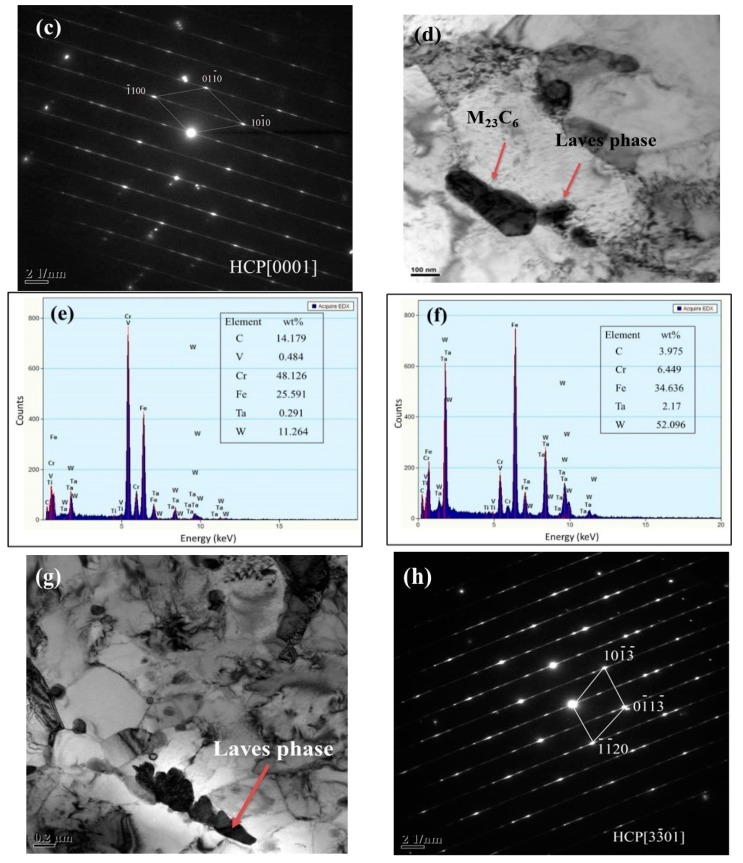
Transmission electron microscopy (TEM) images of CLAM steel for various aging conditions: (**a**) as-received, (**b**) 5000 h, (**c**) selected area electron diffraction (SAED) of the Laves phase for (**b**), (**d**) 10,000 h, (**e**) energy dispersive spectroscopy (EDS) of M_23_C_6_ for (**d**), (**f**) EDS of the Laves phase for (**d**). (**g**) 30,000 h, (**h**) SAED of the Laves phase for (**g**).

The TEM images of the as-received and long-term aged specimens of CLAM steel are shown in Figure 1. The image of the as-received specimen shown in Figure 1a, which clearly exhibits packets, blocks and lath boundaries, shows a typical tempered martensitic microstructure, with fine MX carbonitrides distributed in the matrix and larger sized M_23_C_6_ carbides precipitated at the lath/subgrain boundaries, which play an important role in hindering dislocation movement and boundary migration, effectively improving the stability of the microstructure of the CLAM steel. After aging for 5000 h, the nucleation of the fine Laves phase in the aged sample was detected at subgrain boundaries with a size of approximately 180 nm, as shown in Figure 1b. This was confirmed to be the Laves phase by SAED (Figure 1c). At the same time, the microstructure has a certain recovery, which shows that the lath boundaries begin to be tortuous and widen. 

After aging for 10,000 h, we found two types of precipitates in the aged specimen, as shown in Figure 1d. The EDS analysis shows that the Laves phase mainly attaches to M_23_C_6_ carbide nucleation at the subgrain boundary (Figure 1e–f). This nucleation method can not only reduce the critical nucleation energy but also provide a large number of locations for the Laves phase to nucleate. When the aging time reaches 30,000 h, the martensitic lath has mostly degenerated to form subgrains, the lath boundaries are no longer clear, as shown in Figure 1g, and the Laves phase shows obvious aggregation and coarsening, reaching approximately 800 nm. Figure 1h shows the diffraction pattern of the Laves phase in samples aged for 30,000 h. In the study of aging in the P92 steel, precipitation of the Laves phase at grain boundaries is the main location of crack nucleation, which is easy to cause intergranular cracking, and much W is consumed to reduce the solid solution strengthening [24]. Hence, the coarsened Laves phase in CLAM steel after long-term aging will also lead to a significant decline in the strength, which greatly reduces the stability of the microstructure.

### 3.2. Characterization of Laves Phase Nucleation and Growth during Aging

**Figure 2:**
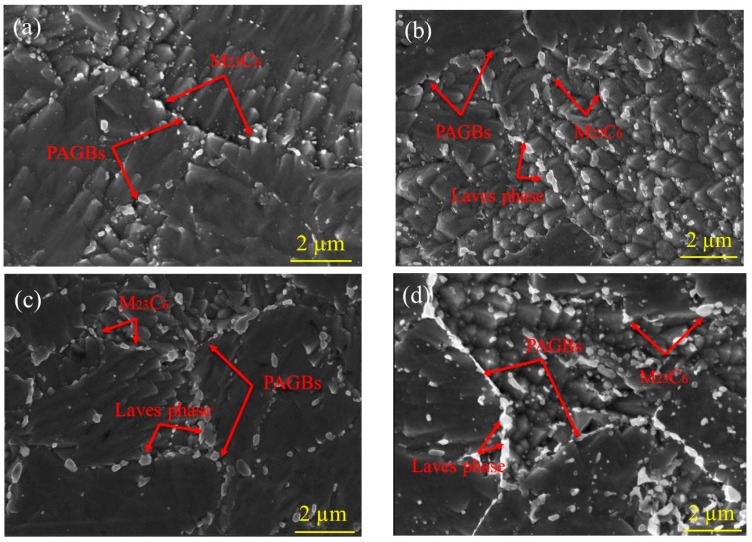
Scanning electron microscopy (SEM) images of CLAM steel for various aging conditions: as-received (**a**), 10,000 h (**b**), 20,000 h (**c**), and 30,000 h (**d**).

Figure 2 shows the secondary electron images of SEM for the as-received and aged specimens of CLAM steel. In the as-received specimen, there are clearly prior austenite grain boundaries (PAGBs) and subgrain boundaries. Fine MX carbonitrides in the matrix and coarse M_23_C_6_ carbides at PAGBs and subgrain boundaries are both noticed, as shown in Figure 2a. After aging for 10,000 h (Figure 2b), due to the formation and growth of the Laves phase, the size of the precipitates at PAGBs and subgrain boundaries increased significantly compared to the as-received specimen. With increasing time, after aging for 20,000 h (Figure 2c), the Laves phase shows a higher coarsening rate, which is expressed by the clear aggregation growth and coarsening of the Laves phase along the PAGBs and subgrain boundaries. 

**Figure 3:**
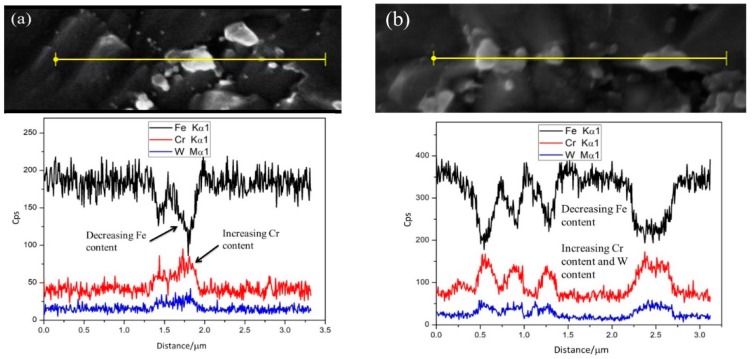

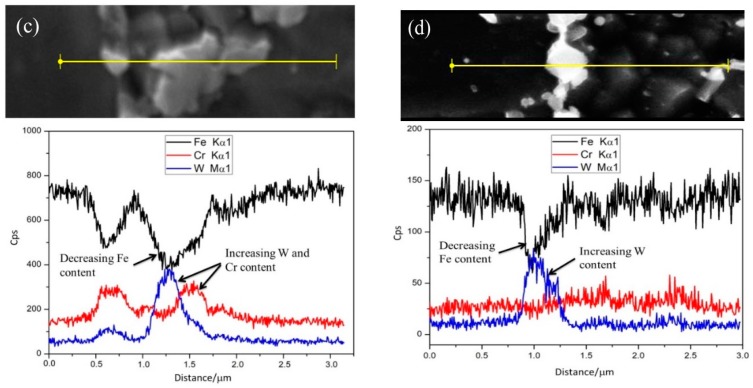
Line mapping results for (**a**) as-received and aged for (**b**) 10,000 h, (**c**) 20,000 h, and (**d**) 30,000 h.

When the aging time reaches 30,000 h (Figure 2d), the coarsened precipitates are distributed in clusters and chains along the PAGBs and subgrain boundaries. In order to confirm this phenomenon is the formation and coarsening of the Laves phase. The line mapping of the as-received and aged specimens was performed as shown in Figure 3, which mainly detects the change of the W content for precipitates at the PAGBs. As W is the main alloying element formed by the Laves phase in CLAM steel, the increase of W content in the precipitate is an important basis for confirming the Laves phase formation. In the as-received sample, as shown in Figure 3a, the line mapping of the precipitate identified a higher content of Cr, which is inferred to be the M_23_C_6_ composition. After aging for 10,000 h (Figure 3b), the content of W and Cr increased, but the Fe content simultaneously decreased in precipitates at PAGBs. As the aging time increases to 20,000 h (Figure 3c), the results of line mapping show that the weight percentage of W and the side of Cr are both clearly increased. It can be concluded that the Laves phase had formed, and corresponding to the morphology of the precipitates, the Laves phase growth and coarsening always depend on the M_23_C_6_ carbides. 

After aging for 30,000 h (Figure 3d), the W content in the precipitate is significantly increased. The Laves phase is obviously coarser than the aging after 10,000 h (Figure 3b), and the size is in the range of 400–500 nm. According to the above results, after long-term aging, it is known that the segregation of the W element to the boundaries is the main reason for the formation of the Laves phase in CLAM steel. The Laves phase always nucleates at grain boundaries by consuming the W element from solid solution, resulting in a reduction of the Laves phase transformation free energy [22]. The experimental phenomena is similar to the studies of Sainia et al. [24] and Panait et al. [25], where the Laves phase in CLAM steel also preferentially nucleates at PAGBs and subgrain boundaries, and exhibits a higher coarsening rate. Coarsening of the Laves phase particles will trigger the formation of voids under creep stress, and subsequently cause brittle intergranular fracture [23]. Hence, the formation of the Laves phase is detrimental to the high temperature mechanical properties of CLAM steel.

**Figure 4:**
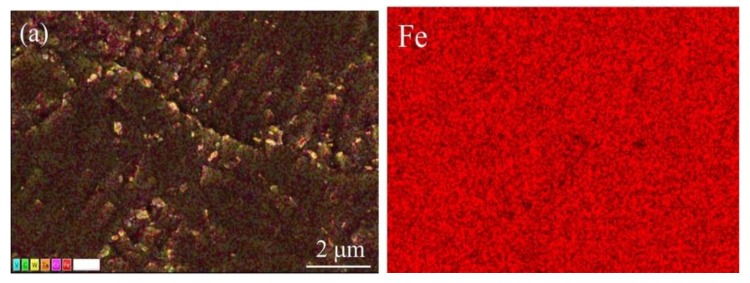

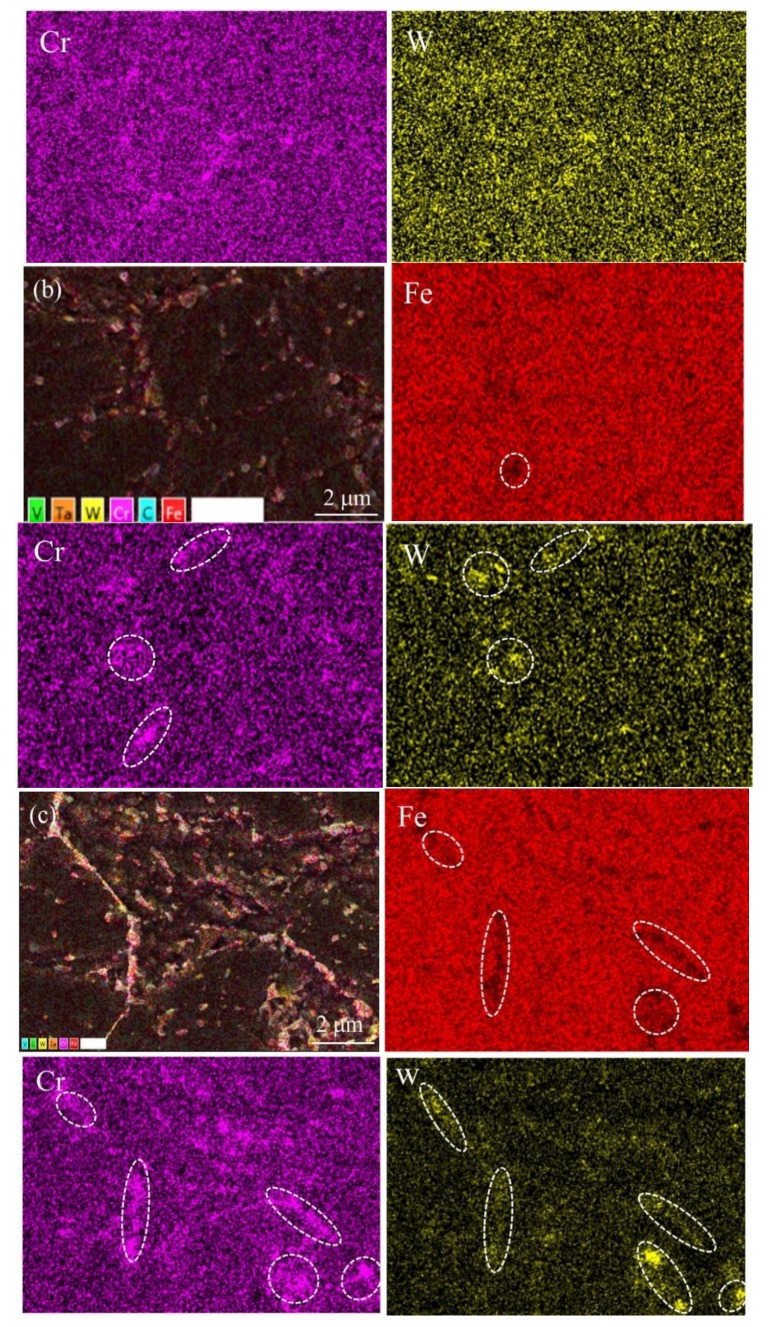
Elemental mapping of CLAM steel in the as-received condition (**a**), after aging of 20,000 h (**b**), and after aging of 30,000 h (**c**).

To better describe the nucleation growth characteristic of the Laves phase in CLAM steel during long-term aging, elemental (Fe, Cr, and W) mapping of as-received and aged samples was also performed, as shown in Figure 4. In the as-received condition (Figure 4a), the Fe and W elements are uniformly distributed; however, some Cr elements are noticeably segregated at the subgrain boundary, which is caused by the presence of Cr-rich M_23_C_6_ carbides in the CLAM steel. With increasing time, in the specimen aged for 20,000 h (Figure 4b), the concentrations of W and Cr increased along the grain boundary, and some Fe contents decreased at the grain boundary. According to the microstructure image, the Laves phase formed and coarsened clearly at the grain boundaries. After aging for 30,000 h (Figure 4c), the aggregation and coarsening of the Laves phase was more serious, and the concentration of Fe decreased obviously along the grain boundary compared to the as-received specimen. 

The segregation position of W was basically consistent with that of Cr, which further confirms that the Laves phase generally nucleates and grows near the M_23_C_6_ carbides at grain boundaries. The nucleation of Laves phase consumes much W in solid solution, resulting in a decrease of the concentration of W in the matrix. In ferritic/martensitic steel, the solid solution strengthening mechanism of W is an important factor to enhance the thermal stability of the material [23]. However, during the long-term aging of CLAM steel, the W atoms in the solid solution will continuously diffuse to the grain boundaries and enhance the formation and growth of the Laves phase, cause W to be consumed continuously. The coarsened Laves phase will weaken the bonding force and the ability of coordinated deformation between the grain boundaries and increase the brittleness of the materials [15]. Finally, the stability of the microstructure declines.

### 3.3. The Evolution of the Laves Phase

**Figure 5:**
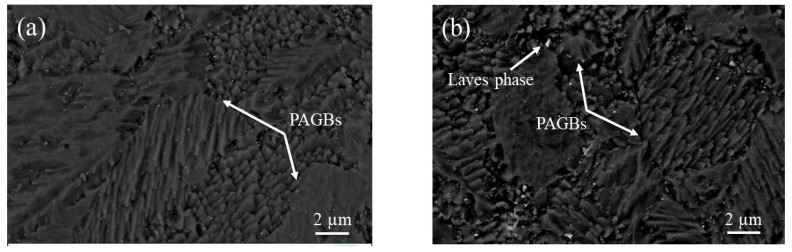

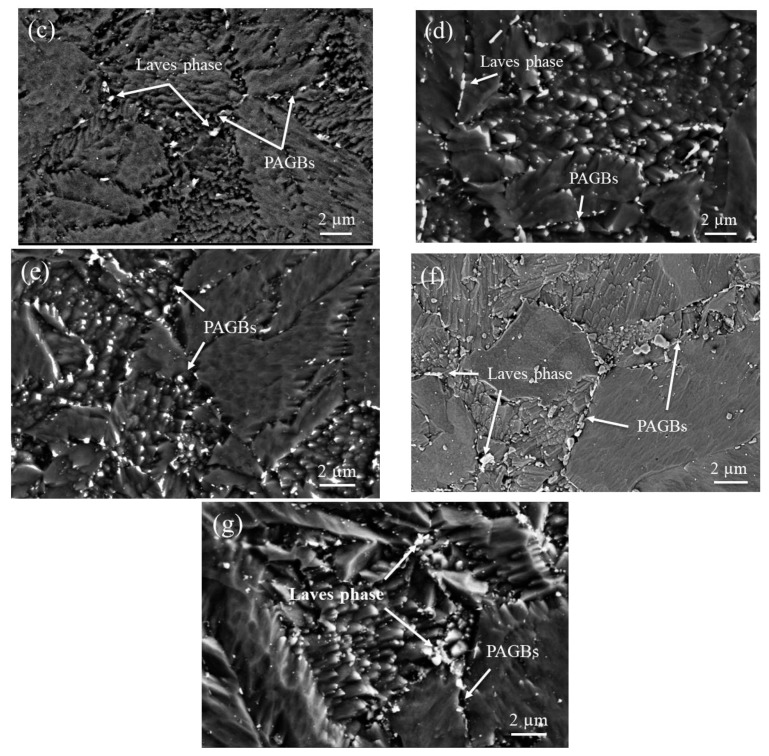
Backscattered electron (BSE) images of CLAM steel as received (**a**) and after aging for 5000 h (**b**), 10,000 h (**c**), 15,000 h (**d**), 20,000 h (**e**), 25,000 h (**f**), and 30,000 h (**g**).

Figure 5 shows SEM-BSE images of the Laves phase evolution at different aging times in CLAM steel. In the as-received sample, we noticed PAGBs and subgrains (shown in Figure 5a). Due to the small average atomic number of M_23_C_6_ carbides, the morphology contrast is not clear. During the aging process, Laves phase particles were observed to nucleate and grow, as shown in Figure 5b–g; when aging to 5000 h (Figure 5b), it was found that the fine Laves phase nucleated at PGABs. After aging for 10,000 h (Figure 5c), the bright Laves phase particles continued to nucleate and grow along the PAGBs and subgrain boundaries. When the aging is 15,000–20,000 h (Figure 5d–e), the Laves phase began to show a joint growth phenomenon. 

After aging from 25,000–30,000 h (Figure 5f–g), the Laves phase particles are aggregated and coarsened, and the number is no longer increased. It gradually reaches saturation after long-term aging, resulting in the number of the Laves phase no longer increasing, only aggregation and coarsening between the particles occurs due to the Ostwald ripening mechanism of the precipitates [16]. Comparing the coarsening mechanism of M_23_C_6_ carbides, Abe [26] proved that M_23_C_6_ coarsening is mainly controlled by the volume diffusion mechanism. However, the Laves phase is not the same in CLAM steel, as the nucleation of the Laves phase is generally at the PAGBs and subgrain boundaries. It is known that the diffusion activation energy of the atom at the grain boundary is lower than that inside the grain, which makes the grain boundary diffusion easier [27]. 

**Figure 6:**
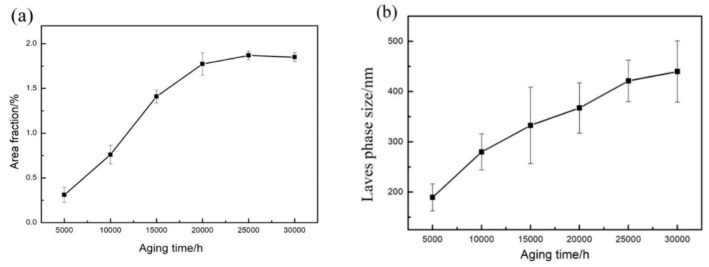
Relationship of aging time on fraction area (**a**) and average size (**b**) of Laves phase.

Therefore, the growth and coarsening of the Laves phase in CLAM steel is mainly controlled by the mechanism of the grain boundary movement. Figure 6 shows a quantitative analysis of the Laves phase based on SEM-BSE images. Figure 6a shows the relationship between the area fraction of the Laves phase and aging time, which shows that the precipitation of the Laves phase increases continuously within 5000–20,000 h. Finally, the area fraction of the Laves phase was stable at approximately 1.85%. Figure 6b shows the change in the average size of the Laves phase particles. Before 25,000 h of aging, the average size increased distinctly, but after 25,000 h of aging, the growth rate of the Laves phase decreased slightly, and the size increase was no longer severe. The average size reached 439.9 nm.

However, in the long-term aging study of F82H steel [17], the Laves phase began to nucleate after aging at 550 °C for 1000 h, which is earlier than the Laves phase in CLAM steel. Hence, the Laves phase in F82H steel quickly reached precipitation saturation after 3000 h of aging, which is stable at approximately 450 nm in the later aging process. The Laves phase shows a higher coarsening rate than CLAM steel. It is believed that the reasonable W content delays the formation of the Laves phase. The W content (1.43%) in CLAM steel is lower than that of F82H steel (W—1.98%). During the aging process, a large amount of W in F82H steel is more likely to be segregated at PAGBs and subgrain boundaries, which causes the Laves phase to be more easily precipitated and coarsened. However, in CLAM steel, the chemical composition is an optimized adjustment, which takes more time to nucleate the Laves phase, and the coarsening rate is lower than that of the F82H steel. Therefore, the microstructural stability of the CLAM steel is improved.

**Figure 7:**
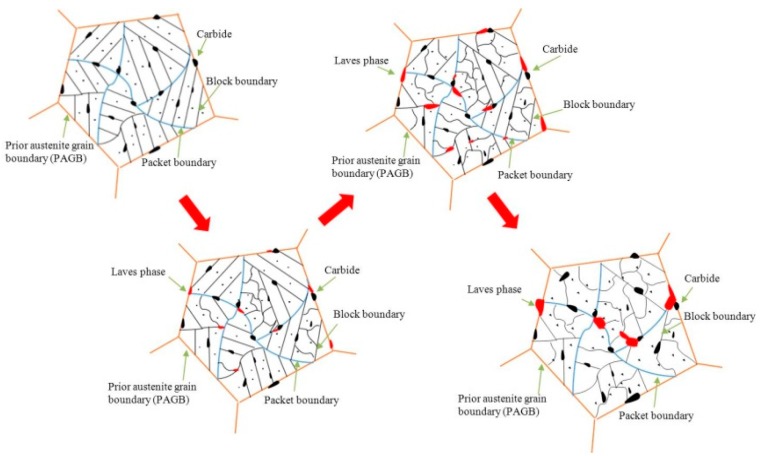
Schematic evolution of the Laves phase.

The nucleation of the Laves phase always depends on the M_23_C_6_ carbide, and is generally precipitated along the PAGBs and subgrain boundaries, as shown in Figure 1d and Figure 5b–c. With prolonged aging time, the Laves phase continued to grow around the M_23_C_6_ carbide and the number also increased continuously, as shown in Figure 3c and Figure 5d–e. At the same time, the martensitic lath gradually degenerates to form subgrains, as shown in Figure 1g. After long-term aging, the precipitation of the Laves phase reaches saturation due to the Ostwald ripening mechanism, which is no longer increased. Only the aggregation and coarsening of Laves phase particles occurs, as shown in Figure 5f–g. The evolution of the Laves phase is divided into three stages: nucleation, growth, and coarsening. To better describe this phenomenon, the evolution is simulated as shown in Figure 7. In the tempered sample, M_23_C_6_ carbides (larger black particles) and MX carbonitrides (fine black particles) are precipitated in CLAM steel. Stage 1: The Laves phase with fine granule shape is mainly nucleated at M_23_C_6_ carbides. Stage 2: The Laves phase continuously grows along the boundaries. Stage 3: The Laves phase has aggregated and coarsened.

## 4. Conclusions

According to the temperature characteristics of the fusion engineering experimental reactor during service, the precipitation and evolution of the Laves phase in CLAM steel over long-term aging at 550 °C was investigated in this paper. The main results are as follows:

(1) The Laves phase does not precipitate in CLAM steel after normalizing and tempering. However, after aging of 5000 h at 550 °C, The Laves phase with fine granule shape was found nucleated along the PAGBs and subgrain boundaries, and it mainly tends to nucleate very close or attached to the M_23_C_6_ carbide.
(2) During the aging process, the continuous diffusion of the W element to the grain boundary is the important factor for improvement of nucleation and growth of the Laves phase. After aging for 30,000 h, much W was consumed in the solid solution, resulting in a decrease in W concentration. At the same time, the coarse Laves phase was distributed in chains and large clusters along the boundary, which greatly weakens the binding force and the ability of coordinated deformation between the grain boundaries.
(3) The growth coarsening of the Laves phase is mainly affected by the mechanism of the grain boundary movement. Before the aging of 20,000 h, the amount and size of the Laves phase continued to increase, but after 25,000 h, the precipitation of the Laves phase reached saturation, due to the Ostwald ripening mechanism. After this point, the number of precipitation no longer increases, only the coarsening of particles occurs. Finally, the area fraction of the Laves phase is approximately 1.85%, and the average size is 439.9 nm. After aging for 20,000 h, the Laves phase was significantly coarsened, resulting in a sharp decrease in the thermal stability of the microstructure. This work completely predicts the safe service life of CLAM steel at the service temperature, which is recommended not to exceed 20,000 h. And it provides the important theoretical guidance for optimizing the microstructure and improving the thermal stability.

## Figures and Tables

**Table 1:** Chemical composition of China low-activation martensitic (CLAM) steel, weight%.

Element	C	Cr	W	V	Ta	Mn	P	S	Fe
wt %	0.091	8.93	1.43	0.19	0.1	0.48	0.05	0.04	Rest
